# Perioperative changes in the plasma metabolome of patients receiving general anesthesia for pancreatic cancer surgery

**DOI:** 10.18632/oncotarget.27956

**Published:** 2021-05-11

**Authors:** Johanna Mock-Ohnesorge, Andreas Mock, Thilo Hackert, Stefan Fröhling, Judith Schenz, Gernot Poschet, Dirk Jäger, Markus W. Büchler, Florian Uhle, Markus A. Weigand

**Affiliations:** ^1^Department of Anesthesiology, Heidelberg University Hospital, Heidelberg, Germany; ^2^Department of Medical Oncology, National Center for Tumor Diseases (NCT) Heidelberg, Heidelberg University Hospital, Heidelberg, Germany; ^3^Department of Translational Medical Oncology, National Center for Tumor Diseases (NCT) Heidelberg, German Cancer Research Center (DKFZ), Heidelberg, Germany; ^4^German Cancer Consortium (DKTK), Heidelberg, Germany; ^5^Department of Surgery, Heidelberg University Hospital, Heidelberg, Germany; ^6^Centre for Organismal Studies (COS), University of Heidelberg, Heidelberg, Germany

**Keywords:** metabolomics, anesthesia, plasma, tumor, longitudinal

## Abstract

Background: Modern anesthesia strives to offer personalized concepts to meet the patient’s individual needs in sight of clinical outcome. Still, little is known about the impact of anesthesia on the plasma metabolome, although many metabolites have been shown to modulate the function of various immune cells, making it particularly interesting in the context of oncological surgery. In this study longitudinal dynamics in the plasma metabolome during general anesthesia in patients undergoing pancreatic surgery were analyzed.

Materials and Methods: Prospective, observational study with 10 patients diagnosed with pancreatic (pre-) malignancy and subjected to elective resection surgery under general anesthesia. Plasma metabolites (*n* = 630) were quantified at eight consecutive perioperative timepoints using mass spectrometry-based targeted metabolomics.

Results: 39 metabolites significantly changed during the perioperative period. Tryptophan concentrations decreased by 45% with the maximum decrease after anesthesia induction (*p* = 6.24E-07), while taurine synthesis increased (*p* = 1.46E-04). Triacylglycerides and lysophosphatidylcholines were significantly reduced with increased liberation of free monounsaturated fatty acids (*p* = 0.03). Carnitine levels decreased significantly (*p* = 9.30E-04).

Conclusions: The major finding of this study was perioperative tryptophan depletion and increased taurine synthesis. Both are essential for immune cell function and are therefore of significant interest for perioperative management. Further studies are needed to identify influencing anesthetic factors.

## INTRODUCTION

Although general anesthesia using intravenous and volatile anesthetics is well and safely established for extended abdominal surgery, little is known about the effects of anesthesia on perioperative metabolism. Few previous studies addressing these issues mainly focused on glucose metabolism, cortisol release and perioperative stress response [1, 2]. However, it is known that also amino acid and lipid metabolism play an essential role for immune performance and inflammatory response [3–5]. While in the past, anesthetic procedures were considered sole prerequisites of surgery, modern anesthesia evolves to become more individualized in an effort to address a patient’s individual needs regarding the metabolic, physiological and immunological integrity in sight of long-term clinical outcome.

This endeavor is of particular importance for surgical oncology conducted under general anesthesia. During surgery, single tumor cells are released into the bloodstream bearing a high potential for further metastatic spread [6]. Since immune cells as natural killer cells or CD8+ T-cells have the capability to identify and eliminate these malignant cells, the perioperative immune status plays an essential role for anti-tumor activities [6–8]. A plethora of factors contribute to a complex modulation of immune function during surgery, e.g., the release of damage-associated molecular patterns (DAMPs) from injured tissue and the organism’s release of metabolites in response to anesthesia and surgical stress. Recently, retrospective studies and meta-analyses indicated that anesthetic procedures (e.g., volatile vs total intravenous anesthesia vs usage of epidural) could affect tumor recurrence and oncologic outcome in cancer patients [9, 10]. There is a hint that intravenous anesthetics (e.g., propofol) may be associated with improved recurrence-free survival rates compared to volatile anesthetics [9, 10]. The underlying mechanisms still remain unclear: impaired natural killer cell activity and t-lymphocyte apoptosis induced by volatile anesthetics observed *in vitro* may be a promising approach [11, 12].

Due to recent technological improvements, it is now possible to quantitatively assess a patient’s plasma metabolome using mass spectrometry. This allows to draw conclusions on individual metabolites, on the current metabolic phenotype and to detect potential deficiencies. For example, certain metabolic constellations can reflect a patient’s immune status (immunosuppressive vs. immunoprotective milieu) or serve as prognostic markers in oncology or cardiovascular diseases [3, 13, 14].

The objective of this prospective, exploratory study is to gain information on longitudinal perioperative alterations in the plasma metabolome during general anesthesia in patients diagnosed with pancreatic malignancies leading to a better understanding for personalized perioperative management.

## RESULTS

### Characteristics of the study group

10 patients (7 males; median age 71 years [range 50–81 years]) were included in the study cohort. Details on the patients’ characteristics are shown in Table 1. Diagnosis of suspected pancreatic malignancy was confirmed postoperatively by board-certified pathologists. 8 patients were postoperatively diagnosed with pancreatic ductal adenocarcinoma (PDAC) and 2 patients with pancreatic premalignancy (intraductal papillary mucinous neoplasm (IPMN) of main duct type). Pylorus preserving pancreaticoduodenectomy was performed in all patients. The most common comorbidities were arterial hypertension (50%) and coronary heart disease (30%).

**Table 1:** Characteristics of the study cohort

**Age** (median years [min; max])	71 [50; 81]
**Male Sex**	7 (70%)
**BMI**, kg/m²	27.5 ± 3.75
**ASA Classification**	
II	5 (50%)
III	5 (50%)
**NYHA Classification**	
I	9 (90%)
II	1 (10%)
**Histologic diagnosis**	
PDAC	8 (80%)
IPMN main duct-type	2 (20%)
**Comorbidities**	
Arterial hypertension	5 (50%)
Coronary heart disease	3 (30%)
Atrial fibrillation	2 (20%)
Other vascular diseases	3 (30%)
**Preoperative fasting period**, *h*	11.3 ± 1.90
**Balanced Anesthesia with**	
Desflurane	7 (70%)
Sevoflurane	3 (30%)
**Combination with Epidural Anesthesia**	6 (60%)
**Duration of**, min	
Total anesthesia	421 ± 87
Anesthesia before surgical procedures	72 ± 15
Surgical procedure	336 ± 86
Surgery until tumor removal	156 ± 81
**Blood loss**, ml	1164 ± 563
**Crystalloid infusion**, ml	3435 ± 1131
**Intraoperative usage of**	
Colloids	5 (50%)
FFPs	3 (30%)
PRBCs	0 (0%)
**Urine volume**, ml	1027 ± 633
**Total amount of**	
Sufentanil, μg	87 ± 42
Propofol, mg	218 ± 116

Data shown as mean ± SD or *N* (%). Abbreviations: BMI: body mass index; ASA: American Society of Anesthesiologists; NYHA: New York Heart Association; PDAC: pancreatic ductal adenocarcinoma; IPMN: intraductal papillary mucinous neoplasm; FFP: fresh frozen plasma; PRBCs: packed red blood cells.

All patients went through a fasting period of approx. 11 h prior to surgery, although only 6 hours were prescribed by the responsible anesthetist.

**Figure 1:**
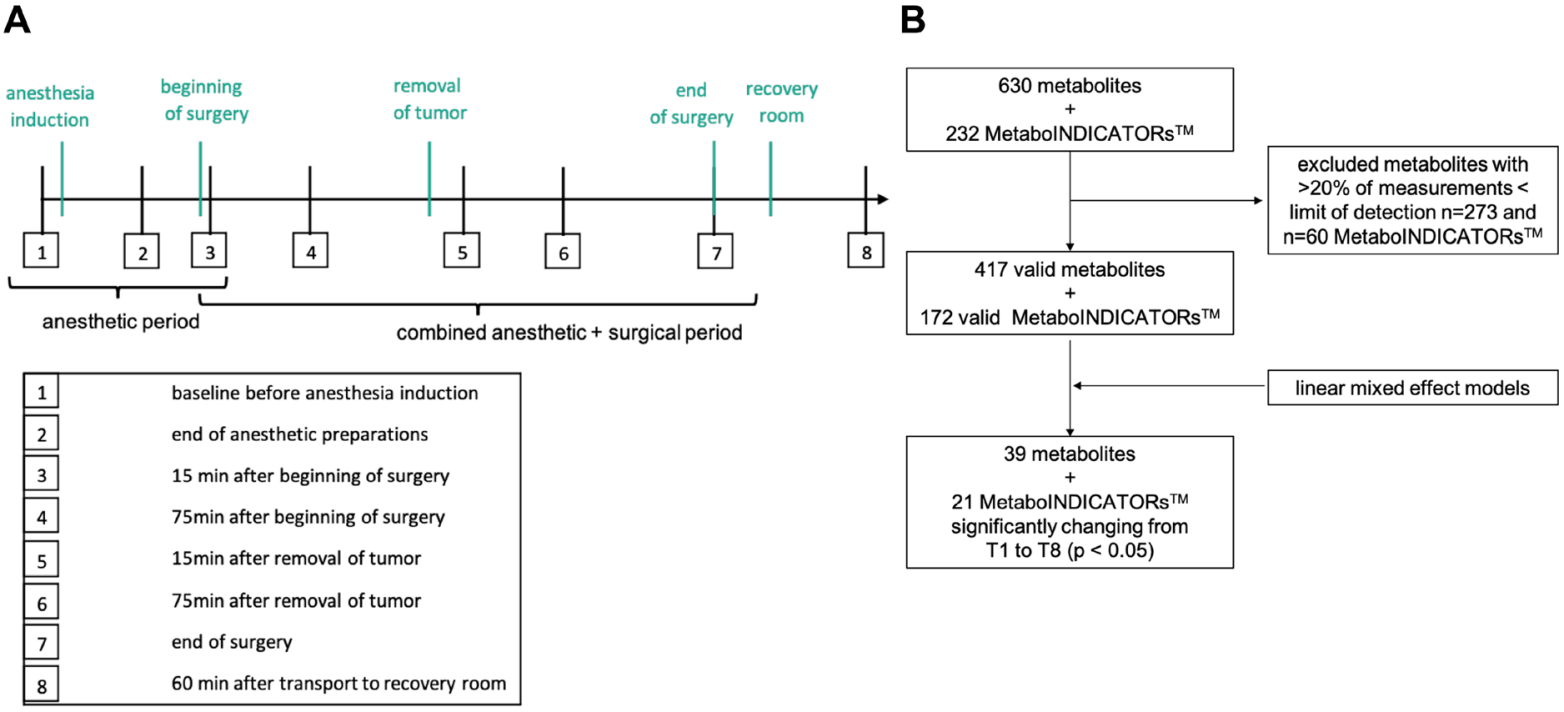
Study design and data processing. (**A**) The diagram shows all 8 timepoints for blood withdrawal within the perioperative period (T1 to T8). Period between T1 and T3 indicates the anesthetic period, T3 to T7 the joint anesthetic and surgical period. Key events are shown in green color. (**B**) Flow diagram showing data processing with exclusion of invalid measurements and identification of significantly changing metabolites during sample timepoint 1 and 8. LOD = limit of detection; T = sampling timepoint; *p* < 0.05. MetaboINICATORS™ are composed ratios or sums of various metabolites allowing further interpretation.

Patients received balanced anesthesia (70% using desflurane, 30% sevoflurane) and 6 patients additionally received epidural anesthesia. Propofol, sufentanil and rocuronium were used for anesthesia induction in all patients. Total duration of anesthesia from anesthesia induction to termination (T1–T7) was 421 ± 87 min including the surgical period (“joint period”) of 336 ± 86 min. This left an observation window of anesthesia related metabolic changes of approx. 72 ± 15 min before beginning of surgery (“anesthetic period”; Figure 1A).

Besides anesthetics every patient received antibiotic prophylaxis within the anesthetic period following the standard regimen using ampicillin/sulbactam. Catecholamines (norepinephrine, cafedrine/theodrenaline) were used both within the anesthetic and joint period in every patient. Norepinephrine infusion reached maximum infusion rates between T4 and T6 (surgical resection period). Dexamethasone was administered in 5 out of 10 patients for PONV prophylaxis. 4 patients received metamizole and 5 patients needed antiemetic drugs (granisetron) at the end of the observation period (T7–T8).

Blood loss was 1164 ± 563 ml and on average 3435 ± 1131 ml crystalloid fluids were infused. Maximum blood loss occurred within 156 ± 59 min between T3 and T5 until the surgical resection phase was completed. There was no transfusion of PRBCs, but 3 patients received fresh frozen plasma for coagulation improvement and 5 patients received colloids (6% hydroxyethyl starch) for cardiocirculatory stabilization.

Postoperative complications included mild symptoms of gastrointestinal dysfunction (*n* = 3), skin erythema (unknown cause; *n* = 1), urinary tract infection (*n* = 1), hypokalemia which required intravenous substitution for three days (*n* = 1), reddening of the injection site of the epidural catheter (*n* = 1), lymphatic fistula (*n* = 1) and chyle fistula (*n* = 1) which required prolonged drainage and anastomosis insufficiency (*n* = 1) which required surgical revision. All patients were dismissed from the hospital in good clinical condition. Wounds healed per primam in all patients. No hematoma, seroma or wound infections occurred.

### Metabolomic data processing

After exclusion of metabolites and MetaboINDICATORS™ with less than 80% valid measurements above the LOD, 417 metabolites (6 acylcarnitines, 1 alkaloid, 1 amine oxide, 20 amino acids and 17 amino acid related metabolites, 8 bile acids, 3 biogenic amines, 3 carboxylic acids, 15 ceramides, 16 cholesterol esters, 1 cresol, 6 diacylglycerols, 1 dihydroceramide, 8 fatty acids, 76 glycerophospholipids, 15 glycosylceramides, 2 hormones, 3 indoles derivatives, hypoxanthine, 14 sphingolipids, 1 sugar, 198 triacylglycerols, 1 vitamin) and 172 MetaboINDICATORS™ remained for further analysis (Figure 1B).

**Figure 2:**
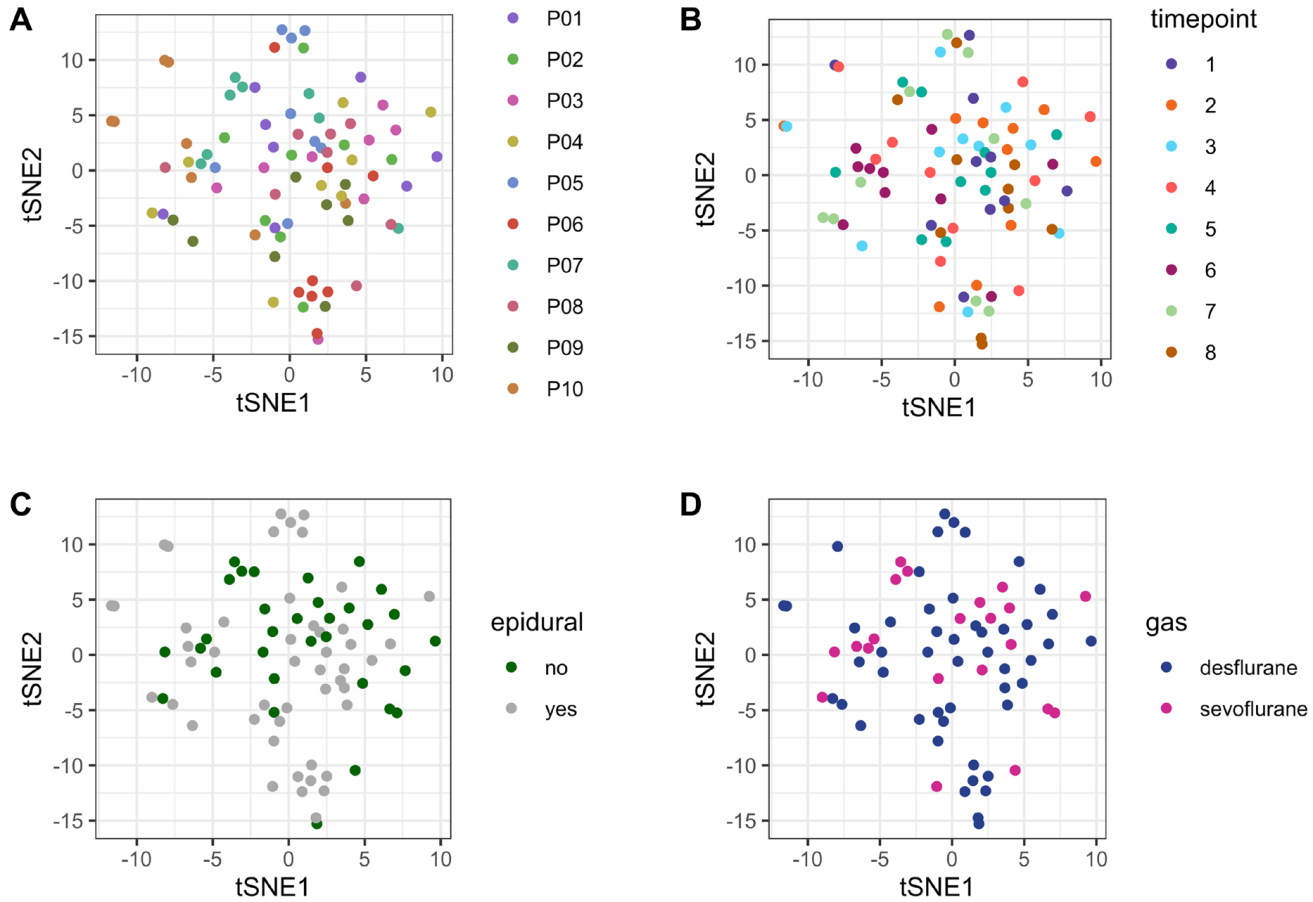
tSNE plot for unsupervised comparison of the metabolome. (**A**) no systematic batch effects were detected within the study cohort grouped by patients. Patients are numbered consecutively (P01–P10). (**B**) no systematic batch effects were detected within the study cohort grouped by sample timepoints, indicating no systematic errors in sample handling/storage. (**C**) Usage of epidural anesthesia had no systematic effect on the plasma metabolome. Metabolome data from all patients and all timepoints (80 samples) are depicted according to whether epidural was used (grey) or not (green). (**D**) There was no systematic effect on the plasma metabolome according to whether desflurane or sevoflurane was applied. Metabolome data from all patients and all timepoints (80 samples) are depicted according to the usage of desflurane (blue) or sevoflurane (violet). Abbreviation: tSNE: t-distributed stochastic neighbor embedding.

There were no systematic differences or batch effects within the metabolome dataset grouped by patients or sample timepoints between patients (Figure 2A and 2B, respectively). Moreover, neither the usage of epidural anesthesia, nor the choice of volatile anesthetic (sevoflurane or desflurane) nor the administration of dexamethasone had a global impact on the patients’ metabolome (Figure 2C and 2D, Supplementary Figure 1, respectively).

Major blood loss occurred during the pancreatic resection period T3–T5. To identify whether blood loss could be responsible for the extent of plasma metabolite concentrations, the per metabolite variance before and after major blood loss was compared. No difference could be observed (*p* < 2E-16; Supplementary Figure 2).

### Perioperative changes in the plasma metabolome

**Figure 3:**
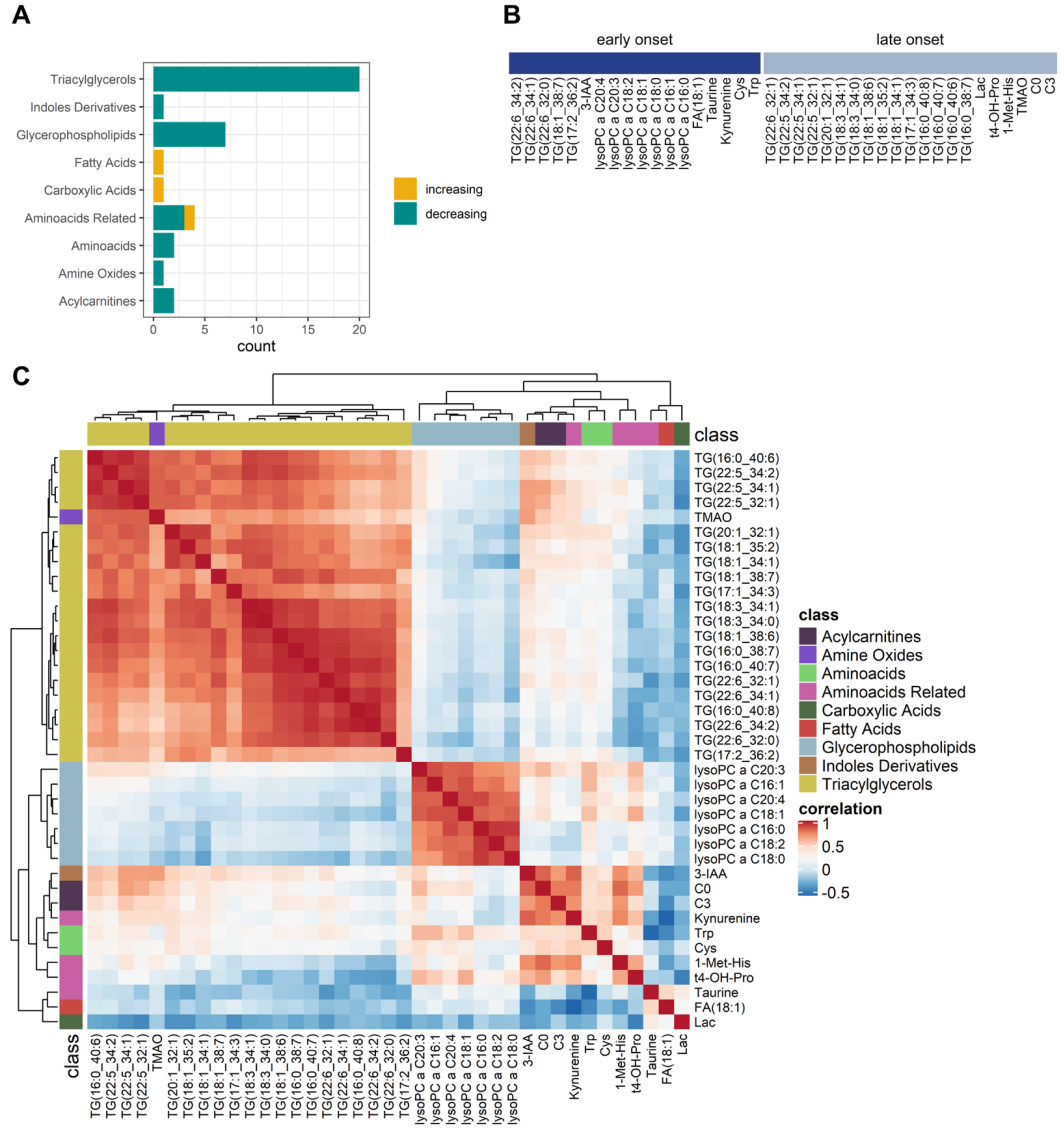
Comparative analysis of perioperative changes in the metabolome. (**A**) Significantly increasing (yellow) and decreasing (green) metabolites grouped by metabolite classes. (**B**) Metabolic dynamics grouped into early onset and late onset changes. Significantly changing metabolites (*n* = 39) were divided into two groups according to whether the absolute coefficient was greater within the anesthetic period than the whole observation period (early onset) or not (late onset). (**C**) Correlation of metabolite alterations: Heatmap. Positively correlated (red) and inversely correlated (blue) metabolites. Lysophosphatidylcholines were strongly correlated within their group. Triacylglycerides were all correlated within their group but formed three subclusters. Carnitines, 3-IAA, and kynurenine were positively correlated. Abbreviations: TG: triacylglyceride; lysoPC: lysophosphatidylcholines; FA: fatty acid; 3-IAA: 3-indoleaceitic acid; Lac: lactate; Cys: cysteine; Trp: tryptophan; t4-OH-Pro: t4-hydroxyproline; 1-Met-His: 1-methylhistidine; TMAO: trimethylamine N-oxide; C0: carnitine; C3: propionyl carnitine. Note that fatty acid residues of triacylglycerides and lysoPCs are denoted as (x:y) or (x:y_n:m) with x as the number of carbon atoms and y as the number of double bonds and n as the total number of carbon atoms and m as the total number of double bonds of two fatty acid residues.

39 metabolites and 21 MetaboINDICATORS™ increased or decreased significantly after adjusting for multiple testing from T1 (baseline before anesthesia induction) to T8 (recovery room) during anesthesia and surgery (*p* < 0.05, adjusted for multiple testing; Figure 1B). Those 39 metabolites included 20 triacylglycerides, 7 lysophosphatidylcholines (lysoPCs, glycerophospholipids), 2 acylcarnitines (carnitine and propionyl carnitine), 2 amino acids (tryptophan and cysteine), 4 amino acid related metabolites (kynurenine, 1-methylhistidine, trans-4-hydroxyproline, taurine), 1 indole derivative (3-indoleacetic acid (3-IAA)) 1 amine oxide (trimethylamine N-oxide (TMAO)), 1 carboxylic acid (lactate) and 1 fatty acid (octadecenoic acid = oleic acid, FA 18:1) (Figure 3A). The individual statistical results of metabolites and MetaboINDICATORS™ are provided in Supplementary Table 1.

Carnitine and propionyl carnitine, tryptophan, kynurenine, cysteine and 3-indoleacetic acid, 1-methylhistidine, trimethylamine N-oxide and trans-4-hydroxyproline as well as various triacylglycerides and lysophosphatidylcholines significantly decreased during the observation period. On contrary taurine, lactate and oleic acid significantly increased from T1 to T8.

Metabolites were grouped into “early onset changes” and “late onset changes”, according to whether the concentration changes (represented by the absolute value of the coefficient of the regression analysis) were greater within T1–T3 than T1–T8 (early onset, starting already within the anesthetic period) or not (late onset, starting within the joint anesthetic and surgical period) (Figure 3B).

Early onset changing metabolites included 5 triacylglycerides, all 7 lysophosphatidylcholines, 3-IAA, oleic acid, taurine, kynurenine, cysteine and tryptophan. Late onset changing metabolites included 15 triacylglycerides, trans-4-hydroxyproline, 1-methylhistidine, trimethylamine N-oxide as well as carnitine and propionyl carnitine. Lactate takes a special position, since there was a remarkable decrease in the early period, followed by an even greater increase in the subsequent period.

Assessing the correlation between the significant metabolites across patients and timepoints revealed that most metabolites are tightly correlated within their metabolic class. In line, while both lysophosphatidylcholines and triacylglycerides decreased over time, they were not positively correlated, underlining their different grouping in early onset (lysophosphatidylcholines) and late onset (majority of triacylglycerides) metabolic changes. Moreover, carnitines and metabolites of tryptophan metabolism (3-IAA and kynurenine) were positively correlated (Figure 3C).

### Early onset metabolomic changes

#### Increased taurine synthesis

**Figure 4:**
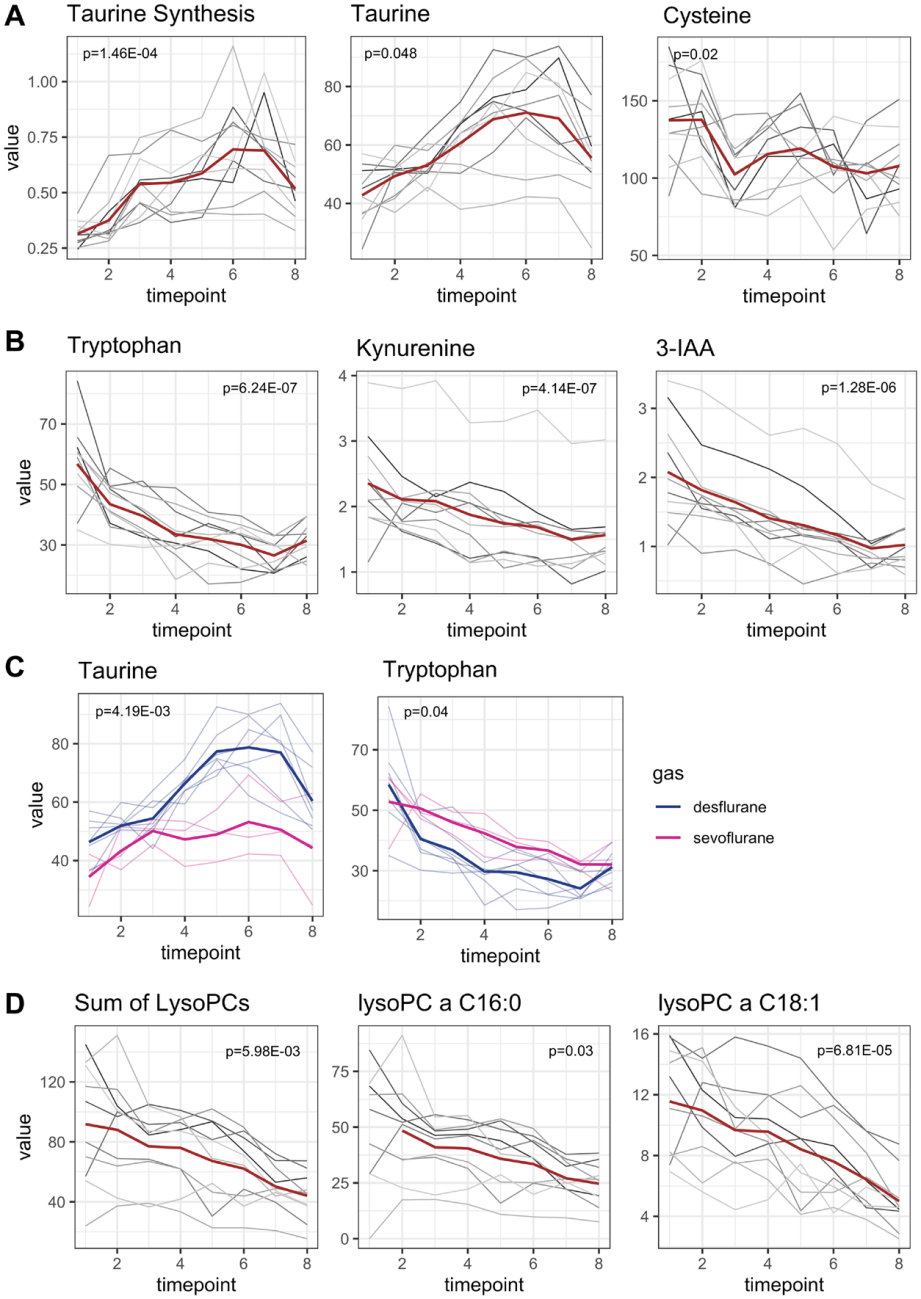
Early onset metabolomic changes. Concentrations are reported in μM. Mean concentrations are depicted as a red line while individual concentration courses of each patient are depicted in grey lines. *p*-values are adjusted for multiple testing. (**A**) Taurine synthesis increased perioperatively with the maximum increase within the anesthetic period (*p* = 1.46E-04). Since taurine is synthesized from cysteine, cysteine concentrations decreased in parallel to taurine increase (*p* = 0.02 and *p* = 0.048, respectively). (**B**) Metabolites related to tryptophan metabolism decreased in parallel to tryptophan decrease. Tryptophan concentration had the strongest decline within the anesthetic period (*p* = 6.24E-07). Metabolites from tryptophan degradation (kynurenine, 3-Indoleaceitic acid) decrease parallelly (*p* = 4.14E-07, *p* = 1.28E-06, respectively). (**C**) Alterations of taurine and tryptophan concentrations were influenced by the choice of anesthetic gas. Patients receiving sevoflurane had lower taurine plasma levels and higher tryptophan levels than patients receiving desflurane (*p* = 4.19E-03 and *p* = 0.04, respectively). *P*-values of multivariable analysis were not adjusted for multiple testing. (**D**) Perioperative metabolism of lysophosphatidylcholines. The sum of all lysophosphatidylcholines decreased significantly (*p* = 5.98E-03). As an example, the two most abundant lysophosphatidylcholines are depicted.

Perioperatively (T1–T8), a significant increase in taurine synthesis (defined as ratio [taurine]/[cysteine]) could be observed (*p* = 1.46E-04, adjusted for multiple testing, MetaboINDICATORS™), characterized with a clear maximum increase within T1 and T3 (coefficient 0.11, *p* = 0.008; Figure 4A). Taurine synthesis out of cysteine started with induction of anesthesia and continued until the end of the observation period. Correspondently, taurine concentrations increased, and cysteine concentrations decreased significantly (*p* = 0.048 and *p* = 0.02, respectively, adjusted for multiple testing). Mean taurine concentrations at baseline (T1) were 42.77 ± 9.96 μM and raised towards 55.56 ± 14.52 μM at the end of the observation period (T8). Mean maximum peak concentrations of taurine were reached at T6 (after removal of tumor, 71.05 ± 16.33 μM) and there was a slight reduction in taurine synthesis afterwards. Mean cysteine baseline concentration was 137.42 ± 30.36 μM and decreased to 107.87 ± 23.02 μM at the recovery room (T8).

#### Tryptophan metabolism

Figure 4B shows metabolites related to tryptophan metabolism. Baseline mean tryptophan concentration was 56.79 ± 14.25 μM. Perioperatively tryptophan concentration was almost halved. At the end of the observation period mean tryptophan concentration at T8 was only 31.47 ± 5.77 μM. Tryptophan decreased immediately and distinctively after anesthesia induction until the end of the observation period (*p* = 6.24E-07, coefficient between T1 and T8 -3.48, Figure 4B). The strongest decrease in tryptophan concentration was seen during anesthesia induction (coefficient between T1 and T3 –8.61).

Indolamin-2,3-dioxygenase activity (IDO-activity), defined as ratio of kynurenine synthesis out of tryptophan remained stable over time (Supplementary Figure 3A). Kynurenine concentrations even decreased in parallel to tryptophan decrease (*p* = 4.14E-07, adjusted for multiple testing; coefficient –0.12, Figure 4B) with mean baseline kynurenine concentration and final concentration at T8 of 2.35 ± 0.76 μM and 1.56 ± 0.56 μM, respectively. 3-Indoleacetic acid (3-IAA), a metabolite of tryptophan metabolism in gut microbiota, was also halved during observation period with mean baseline concentration 2.08 ± 0.78 μM and final concentration 1.02 ± 0.34 μM (*p* = 1.28E-06, adjusted for multiple testing, Figure 4B). In the multivariate analysis, administration of dexamethasone was associated with higher 3-IAA concentrations compared to patients who did not receive dexamethasone (*p* = 9.16E-03). Serotonin synthesis, which also requires tryptophan, remained beyond the limit of detection.

In the multivariate analysis, the choice of anesthetic was identified as an influencing factor of taurine and tryptophan plasma concentrations. Patients receiving desflurane had higher taurine levels but lower tryptophan levels than patients receiving sevoflurane (Figure 4C, *p* = 4.19E-03 and *p* = 0.04, respectively). Mean average taurine concentrations during the observation period (T1–T8) were 45.52 ± 10.72 μM and 64.06 ± 14.580 μM for sevoflurane and desflurane, respectively. Accordingly, mean average tryptophan concentrations were 41.31 ± 9.46 μM for sevoflurane and 34.69 ± 12.76 μM for desflurane. However, tryptophan concentrations were almost equal at the end of the observation period between both groups.

#### Glycerophospholipids

7 lysophosphatidylcholines significantly decreased from T1 to T8 (*p*-values < 0.04, adjusted for multiple testing, Figure 4D). The fatty acid residues of all detected lysophosphatidylcholines were long chained fatty acids (more than 14 carbon atoms) and most were unsaturated. For interpretation, the sum of all lysophosphatidylcholines was used. Lysophosphatidylcholines decreased significantly (*p* = 5.98E-03), especially during the early onset period. The summed concentration of all lysophosphatidylcholines (*n* = 8) decreased from 91.8 ± 40.53 μM to 44.05 ± 16.04 μM. The two most abundant lysophosphatidylcholines (lysoPC a C 16:0 and lysoPC a C18:1) are depicted as examples for the concentration courses (Figure 4D).

In a multivariate analysis, the administration of dexamethasone was identified as an influencing factor for 5 of the 7 lysophosphatidylcholines. Patients receiving dexamethasone for PONV prophylaxis had higher plasma levels of lysophosphatidylcholines compared to those who did not receive dexamethasone (*p* < 0.04).

### Late onset metabolomic changes during joint surgical and anesthetic period

#### Carnitines and b-oxidation

**Figure 5:**
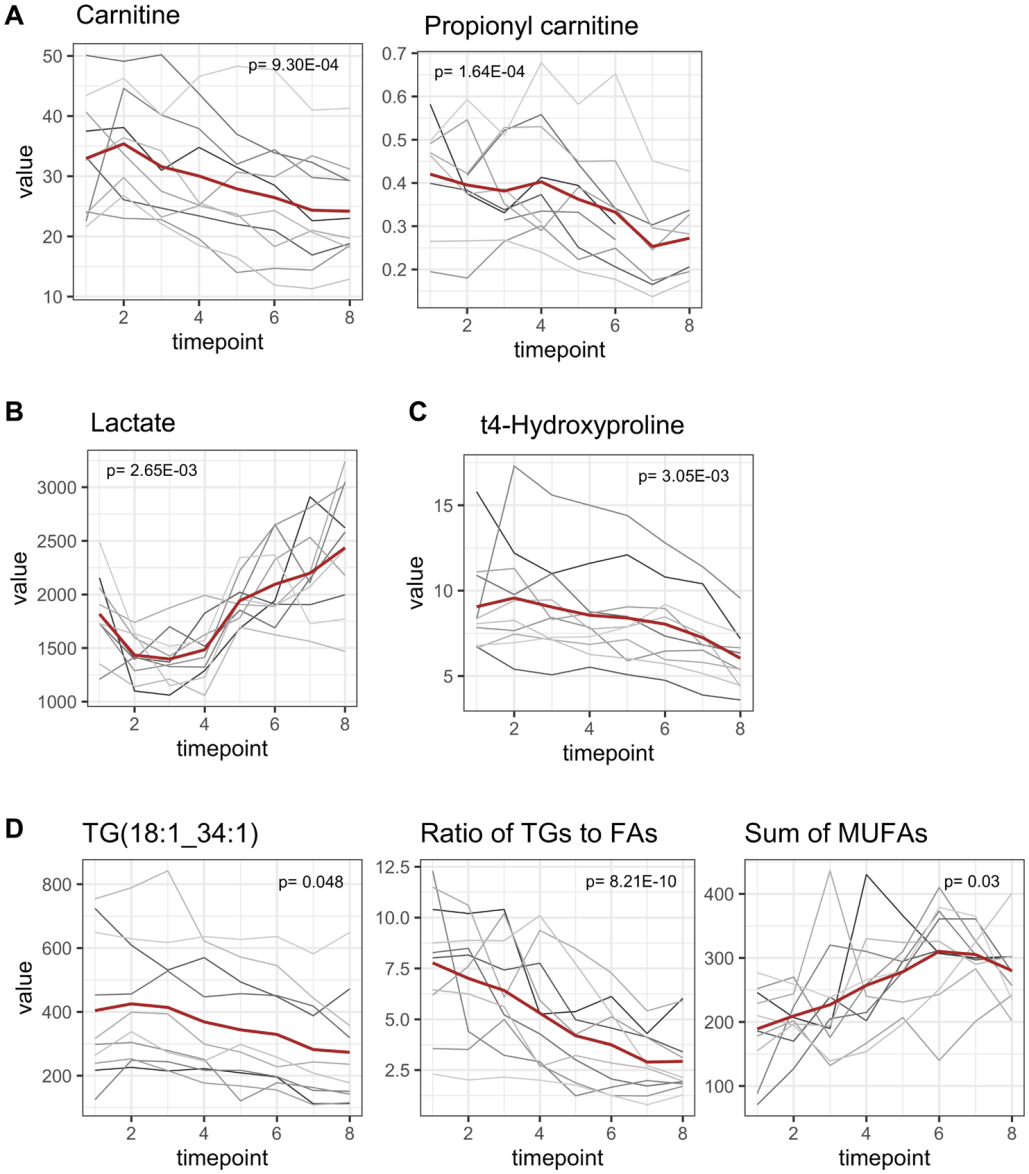
Late onset metabolomic changes. All concentrations are reported as μM. Mean concentrations are depicted as a red line while individual concentration courses of each patient are depicted in grey lines. *p*-values are adjusted for multiple testing. (**A**) Carnitine decreased significantly from T1 to T8 (*p* = 9.30E-04, adjusted for multiple testing) and propionyl carnitine decreased to a slighter extent (*p* = 1.64E-04). (**B**) Highly dynamic alterations in lactate concentrations. Lactate concentrations decreased within the anesthetic period and increased to an even greater extent in the consecutive joint period (*p* = 2.65E-03). (**C**) Hydroxyproline (marker for collagen turnover) significantly decreased after beginning of surgical procedures (*p* = 3.05E-03). (**D**) Triglyceride concentrations decreased in perioperative period. Triacylglyceride 18:1_34:1 as the most abundant one is presented exemplarily. The ratio of [triacylglycerides]/|fatty acids] serves as an indicator for synthesis of triacylglycerides and decreased, symbolizing a degradation of triacylglycerides (*p* = 8.21E-10). Sum of fatty acids in plasma increased, although this was only significant for monounsaturated fatty acids (*p* = 0.03).

Carnitine decreased from 32.95 ± 9.91 μM to 24.20 ± 8.44 μM at the end of the observation period with a maximum decrease in the joint period T3–T8 (*p* = 9.30E-04, adjusted for multiple testing, coefficient T1–T8 = –1.60) (Figure 5A). To a slighter degree, propionyl carnitine also decreased. In the multivariate analysis, the administration of dexamethasone was identified as an influencing factor for carnitine levels and was associated with higher carnitine levels compared to patients who did not receive dexamethasone (*p* = 9.6E-04).

Alterations in carnitine concentrations are related to b-oxidation of fatty acids since carnitine serves as a shuttle to transport activated fatty acids (Acyl-CoA) from the cytosol into the mitochondrial matrix. The ratio of propionyl carnitine plus acetyl carnitine to carnitine ([propionyl carnitine + acetyl carnitine]/[carnitine]), serves as an indicator for b-oxidation rates. Baseline ratio for b-oxidation was 0.27 ± 0.10 and increased to 0.35 ± 0.10 but lost statistical significancy after adjusting for multiple testing (Supplementary Figure 3B).

#### Lactate concentrations

Lactate concentrations were highly dynamic. During the anesthetic period lactate concentrations decreased but increased afterwards during the joint period to an even greater extent (Figure 5B). Therefore, lactate concentrations were classified as late onset metabolomic changes. Baseline lactate concentration was 1817.6 ± 371.6 μM. Minimum and maximum lactate concentrations were reached at T3 and T8, with 1397 ± 248.02 μM and 2435.3 ± 583.43 μM, respectively. Lactate concentrations were higher at the end of the observation period compared to baseline.

#### T4-hydroxyproline: an indicator for collagen turnover

T4-hydroxyproline is a translational modified version of proline and derives from collagen degradation and is therefore associated with tissue damage or collagen turnover. However, after beginning of surgical procedures plasma levels of hydroxyproline decreased from 9.06 ± 2.85 μM to 6.04 ± 1.77 μM (Figure 5C; *p* = 3.05E-03, adjusted for multiple testing).

In addition, the administration of dexamethasone was identified as an influencing factor of t4-hydroxyproline plasma concentrations (*p* = 9.0E-03). Dexamethasone was associated with higher hydroxyproline levels compared to patients who did not receive dexamethasone.

#### Triacylglycerides

The majority of all altered triacylglycerides (*n* = 20, triacylglyceride 18:1_34:1 as the most abundant one is given as an example, Figure 5D) decreased within the joint period as late onset, although 5 already started to decrease earlier (Figure 3B). It must be noted that due to technical aspects isobars of all listed triacylglycerides cannot be distinguished. In summary at least 7 triacylglycerides contained very long chain fatty acid residues (≥ 22 carbon atoms) and when taking potential isobars into account, 3 more (16:0_40:8; 16:0_40:7; 16:0_40:6) are very likely to contain a long chain fatty acid residue [15].

Most triacylglycerides were high in polyunsaturated fatty acids, containing up to 8 double bonds within 3 fatty acid residues.

The ratio of [triacylglycerides]/[fatty acids] decreased from T1 to T8 (*p* = 8.21E-10, adjusted for multiple testing), indicating possible degradation of triacylglycerides with liberation of fatty acids in context of lipolysis. Accordingly, the sum of monounsaturated fatty acids increased over time (*p* = 0.03, adjusted for multiple testing), although the increase of the sum of all fatty acids (saturated, mono- und polyunsaturated) did not reach statistical significance after adjusting for multiple testing.

## DISCUSSION

In this prospective study, we detected perioperative plasma dynamics of metabolites in patients undergoing anesthesia for pancreatic surgery. Due to the longitudinal study setup, we were able to outline metabolic dynamics in a clinical setting, covering the period from anesthesia induction until transfer to the recovery room.

Shortly after anesthesia induction, first significant changes within the plasma metabolome with a focus on amino acids were revealed, emphasizing that anesthesia-associated metabolic alterations exist independently from surgical stress.

### Tryptophan metabolism

Perioperative tryptophan depletion is one of the major findings of this study. Since tryptophan is an essential amino acid, it is of special significance. Baseline tryptophan concentration in the study cohort was 56 μM. This is in line with previous results using the same technology, reporting tryptophan plasma concentrations to be 63 μM [16].

Tryptophan decrease was most pronounced directly after anesthesia induction but was maintained over the whole observation period. Tryptophan can be metabolized via hepatic tryptophan-2,3-dioxygenase (TDO) and the ubiquitarily available indoleamine 2,3-dioxygenase (IDO) into kynurenine and its further metabolites [17, 18]. To a lesser extent tryptophan is used for serotonin synthesis or metabolized by gut microbiota leading to measurable 3-IAA/3-IPA (indole derivatives) concentrations in blood plasma [19, 20].

Moreover, it is known that tryptophan is essential for the immune system, especially for T-cell function: Tryptophan starvation stops T-cell proliferation and differentiation, leading to an immunosuppressive microenvironment [21, 22]. This phenomenon is utilized by cancer cells: they induce overexpression of IDO to promote tryptophan degradation via the kynurenine pathway [4, 5, 23]. Since PDAC is known for its low immunogenicity with a immunosuppressive tumor microenvironment and lacking T-cell infiltration, further perioperative immunosuppression may aggravate these circumstances [24]. Tryptophan depletion is also observed under inflammatory conditions, indicating that negative feedback mechanisms may play a role in tryptophan metabolism and immune effects [25, 26].

Interestingly, all metabolites related to tryptophan catabolism decrease parallelly to tryptophan degradation. This indicates that neither the kynurenine pathway nor serotonin pathway can explain tryptophan degradation. Similarly, 3-indoleaceitic acid (3-IAA), a metabolite of intestinal tryptophan metabolism also decreased, revealing that tryptophan is no longer available for further intestinal metabolism. Complementary, impaired gastrointestinal perfusion might also be responsible for a lower uptake into the blood stream. Perioperative tryptophan decrease might be explained by increased tryptophan consumption by e.g., immune cells or other compartments.

Further investigations are needed including metabolome analysis of feces, urine and immune cells to find sufficient explanation for perioperative tryptophan depletion and whether supplementation of tryptophan might be beneficial for long-term clinical outcome.

### Taurine synthesis

Taurine plasma concentrations, a non-proteinogenic amino acid, increased after anesthesia induction. Taurine supply is mainly provided via dietary intake, but taurine can also be synthesized from cysteine by cysteine dioxygenase and cysteine-sulfinate decarboxylase [27]. Since cysteine concentrations decreased inversely to taurine increase, we assume that taurine increase is indeed caused by higher synthesis rates. Taurine plays an essential role in anti-oxidant, anti-inflammatory and membrane stabilizing processes [27, 28]. This explains its inhibitory effects on cancer cell growth [29]. Moreover, taurine deficiency is linked to type II diabetes, cardiovascular diseases and obesity while taurine supplementation lowers arterial blood pressure and has positive effects on insulin sensitivity [27, 28, 30, 31].

Since taurine concentration is known to be considerably high in neutrophil granulocytes, it is assumed that taurine is also essential for immune function and may be associated with inflammation caused by oxidative stress [32]. This is underlined by the observation that taurine is depleted in patients suffering from severe septic shock and immunological dysregulation [33].

Perioperatively increased levels of taurine may be expression of an effective resource mobilization needed during perioperative inflammation to maintain cellular homeostasis. The increased need for taurine synthesis can be sufficiently covered during anesthesia and surgery in this study cohort and may even be stimulated after anesthesia induction, since taurine synthesis increased steepest directly after anesthesia induction.

### b-oxidation and energy supply

Average baseline levels of carnitine were 32.05 μM in our study cohort which is in line with previous described levels after an overnight fasting period [16, 34]. Alterations in carnitine are related to b-oxidation of fatty acids since free carnitine serves as a shuttle to transport activated fatty acids (Acyl-CoA) from the cytosol into the mitochondrial matrix [35]. Under physiological conditions, oxidation of fatty acids supplements glucose-derived energy supply. However, there are tissue specific preferences: brain tissue is almost entirely dependent on glucose as energy source, while cardiomyocytes use up to 70% of their energy supply from fatty acid oxidation [35].

The ratio of acyl carnitines (predominately acetyl carnitine and propionyl carnitine) and free carnitine serves as an indicator for b-oxidation rates [36]. Normal ratios of acylcarnitine and free carnitine are described to be around 0.25, which was in line with our results [37]. Overnight fasting period seems to not have altered standard carnitine ratios. There was a not statistically significant increase in b-oxidation rates with a later onset, shortly after the beginning of surgery. This is in contrast to reports from a previous study in patients undergoing bariatric surgery, where perturbed mitochondrial metabolism by propofol infusion was suggested to attenuate b-oxidation [38].

Makrecka et al. showed that plasma concentrations of carnitine and acylcarnitines are comparable with energy profiles of the heart muscle [39]. Since skeletal muscles are mainly resting during anesthesia (usage of muscle relaxants) our results might at least reflect an increased perioperative carnitine consumption for b-oxidation in heart muscle cells.

### Lactate

Normal plasma lactate levels are described to be < 2.4 mmol/l. In this study cohort baseline levels were at the upper limit with 2.08 mmol/l in average. This might be due to the 12 hour fasting period before surgery leading to less fluid intake and worsened metabolic and rheologic conditions. Course progression of lactate concentrations has been studied in various clinical settings before, especially in major cardiac surgery [40]. Increase in lactate concentration can be caused by impaired tissue perfusion [40–42]. Relevant blood loss starts with proceeding surgery resulting in hypotension, vasoconstriction and centralization accompanied by intensified catecholamine therapy. Microcirculation is worsened leading to an increasing lactate production from anaerobic glycolysis due to impaired oxygen and metabolic supply [42]. For crystalloid infusion, balanced solutions were used, not containing relevant lactate concentrations.

There is also a remarkable decrease in the very beginning of the observation period during the anesthetic period. After a fasting period of approx. 12 hours, first crystalloid infusions beginning with anesthesia induction may lead to improved rheologic conditions with better peripheral organ perfusion, better oxygen and metabolic supply and subsequently decreased lactate concentrations.

### Hydroxyproline

Hydroxyproline is a post translationally modified form of the amino acid proline, used for collagen synthesis and serves as a marker for collagen turnover [43, 44]. After beginning of surgical procedures, plasma hydroxyproline concentrations decreased despite tissue damage and assumed collagen release. This might indicate that surgical tissue manipulation stopped collagen turnover or that free hydroxyproline is excreted e.g., via urine. Venbrocks et al. also described decreased plasma hydroxyproline levels directly after surgery but plasma concentrations increased after the 3rd postoperative day [45]. Since there is evidence that supplementation of hydroxyproline and collagen hydrolysates may improve wound healing [44, 46], further studies are necessary to evaluate the further postoperative concentrations of hydroxyproline to determine whether supplementation of hydroxyproline might be a useful tool to improve postoperative wound healing. In this study cohort all wounds healed per primam.

### Lipids

Triacylglycerides decreased perioperatively. Triacylglycerides are the storage form of fatty acids and degradation is strictly regulated by hormone-sensitive lipase. Together with an increase in monounsaturated fatty acids coupled with a decrease in plasma carnitine levels, it supports the hypothesis that free fatty acids are used for b-oxidation and energy supply.

Lysophosphatidylcholines also decreased perioperatively. Lysophosphatidylcholines in plasma mainly derive from phosphatidylcholines or lipoproteins. Metsuda et al. reported that decreased lysophosphatidylcholine levels were associated with postoperative inflammation and complications [47]. In healthy subjects, lysophosphatidylcholine concentrations are reported to be around 200–300 μM [48], while in our cohort the sum of all measured lysophosphatidylcholines was only 91.8 μM. Reduced lysophosphatidylcholine levels in cancer patients were associated with inflammatory processes and weight loss [48].

### Limitations

There are limitations to this study, first of all the small sample size. We decided to first investigate metabolomic alterations within a small sample group to identify appropriate timepoints for measurements during the perioperative period for further analysis. For future studies, measurement of four perioperative timepoints (baseline, end of anesthetic preparations, tumor removal, end of surgery) are probably sufficient, complemented by measurements within the postoperative days.

Moreover, we only measured blood plasma concentrations and cannot identify metabolic shifts into other compartments. Further analyses would benefit from measuring also urinary and tissue metabolomics to trace metabolite fluxes.

### Perspectives

Metabolomics represent a phenotype of all genetic, transcriptomic and posttranslational modifications. Perioperative metabolomic analysis enables to get a more accurate insight on perioperative processes. This will allow to answer individual needs enabling personalized anesthetic management. In sight of surgical oncology, it might be even possible to identify therapeutic windows with best conditions for perioperative immunotherapy or chemotherapy. Further controlled studies are desperately needed to compare the impact of different anesthetic procedures on the plasma metabolome to identify beneficial or detrimental influences on long-term clinical outcome.

## MATERIALS AND METHODS

### Study design

In this prospective, non-interventional, monocentric study, 10 patients receiving general anesthesia for pancreatic cancer surgery (planned pylorus preserving pancreaticoduodenectomy (PPPD)) between 04/2019 and 08/2019 were included. PPPD was chosen because of its sufficient length of operation and well-defined surgical steps which allows to monitor metabolomic changes over an adequate period of time and to determine unified perioperative timepoints for blood sampling. Surgery was performed at the Department of General Surgery, Heidelberg University Hospital.

Inclusion criteria were age ≥ 18 years and indication for PPPD due to high suspicion of pancreatic head malignancy under general anesthesia. Exclusion criteria were neoadjuvant chemotherapy (< 3 months), blood transfusion (< 14 days) before surgery, renal insufficiency (glomerular filtration rate GFR < 60 ml/min), pharmacologically treated diabetes mellitus type 1 or 2, autoimmune disease or primary immunodeficiency, immunosuppressive medication and mental disorders. All patients were informed about the study procedures and gave informed consent prior to study enrollment. The study was approved by the local ethics committee of the University of Heidelberg (S-860/2018) and registered at DRKS (German Clinical Trial Register, DRKS00016686).

For plasma metabolome analysis blood was drawn at eight predefined timepoints during general anesthesia and surgery from each patient (Figure 1A). The first blood sampling was scheduled right before the start of general anesthesia induction (baseline) after an overnight fasting period, the second withdrawal after anesthesia induction and preparation (but before beginning of surgery), the third and fourth blood sampling were set 15 min and 75min after beginning of surgery, respectively. The fifth and sixth blood samples were drawn 15 min and 75 min after removal of the tumor and the seventh blood sampling was scheduled at the end of surgery. The final blood sample was drawn 60min after transport to the recovery room. Period T1–T3 includes mainly the anesthetic period without surgical interventions, while T3–T7 spans the joint anesthetic and surgical period.

### Endpoints

The primary endpoint of this study was to detect longitudinal perioperative plasma metabolome changes in patients receiving general anesthesia for oncologic pancreatic surgery. The secondary endpoints were identification of anesthetic influences on the plasma metabolome in context of immunologic consequences for oncologic patients undergoing general anesthesia and to derive hypotheses for further anesthetic studies on influence of anesthesia on oncological outcome.

### Sample handling and targeted metabolome analysis

Within 40 min after withdrawal, blood samples were centrifuged for 10 min at 2500× g and 20°C and separated plasma was frozen and stored at –80°C until mass spectrometry measurement (LC-MS/MS). Storage time at –80°C until mass spectrometry analysis was ≤ 4 months for all samples. For mass spectrometry measurements all samples were transferred to the collaborating laboratory at the Centre for Organismal Studies (COS) Heidelberg.

The MxP Quant 500 Kit by Biocrates Life Science AG, Innsbruck, Austria was used for targeted metabolomic analysis. The MxP Quant 500 Kit enables quantification of up to 630 different metabolites from 26 compound classes (e.g., amino acids, bile acids, biogenic amines, fatty acids, hormones, acylcarnitines, (lyso-)phosphatidylcholines, sphingomyelins, ceramides, cholesteryl esters, di-/triglycerides). Lipids and hexoses were measured by flow injection analysis-tandem mass spectrometry (FIA-MS/MS) and small molecules were measured by liquid chromatography-tandem mass spectrometry (LC-MS/MS). Quantification of the plasma metabolites was achieved by using internal standards. In summary, a 96-well based sample preparation device was used according to the manufacturer’s guidelines. This device consists of inserts that have been impregnated with internal standards, and a predefined sample amount was added to the inserts. Next, a phenyl isothiocyanate (PITC) solution was added to derivatize some of the analytes (e.g., amino acids), and after the derivatization was completed, the target analytes were extracted with an organic solvent, followed by a dilution step. The obtained extracts were then analyzed by FIA-MS/MS and LC-MS/MS methods using multiple reaction monitoring (MRM) to detect the analytes.

Biocrates Life Science AG provided metabolomic summary features termed “MetaboINDICATOR™”, which consisted of ratios and sums of the measured 630 metabolites, aiming to reveal biochemical pathways and contexts. In total 232 MetaboINDICATORs™ were part of the analysis.

All concentrations of plasma metabolites are reported in μM. Concentrations of metabolites are addressed with square brackets as “[metabolite]” in the text. Notation for metabolites as lipids, phospholipids and fatty acids were adopted from Biocrates Life Science AG, Innsbruck, Austria. Note that fatty acid residues of triacylglycerides or lysophosphatidylcholines are denoted as (x:y_n:m) with x as the number of carbon atoms and y as the number of double bonds of the first fatty acid residue and n as the total number of carbon atoms and m as the total number of double bonds of two fatty acid residues. A list of all isobars is provided by Biocrates Life Science AG, Innsbruck, Austria [15].

### Data collection

Patients’ characteristics, routine anesthetic assessment (ASA and NYHA classification, medical condition and medication, allergies, consumption of tobacco/alcohol, etc.), previous oncologic treatment and routine laboratory parameters were collected from the patients’ records. In addition, dose, type and time of applied anesthetic medication, supportive medication (e.g., PONV prophylaxis), antibiotics, blood loss, infused volume, blood transfusion, urinary volume, vital and ventilation parameters and parameters from blood gas analysis were collected. Additionally, surgical parameters as resection margins, TNM classification, results from histological examination of the removed tissue and postoperative complications were collected.

### Anesthesia conduction

The choice of anesthetic medication and procedure remained with the responsible anesthetist who was not part of the study team. In general, internal standards for administration of anesthesia for pancreatic surgery at Heidelberg University Hospital were followed and in absence of contraindications propofol, sufentanil and rocuronium were used for anesthesia induction followed by balanced anesthesia (sevoflurane or desflurane, supplemented by additional bolus injections of sufentanil or rocuronium if necessary). Every patient received arterial blood pressure measurement, a central venous catheter, urinary catheter and gastric tube. Epidural anesthesia using ropivacaine was offered complementarily.

### Statistical analysis

The statistical analyses were conducted using R (R version 4.0.2.; https://www.r-project.org/). Quantitative metabolomic analyses were performed by the Metabolomics Core Technology Platform at the Centre for Organismal Studies (COS) Heidelberg using the MxP Quant 500 Kit by Biocrates Life Science AG, Innsbruck, Austria. Metabolites with more than 20% of all measurements smaller than the limit of detection (LOD) were excluded. The LOD has been defined as < 3× signal to background noise peak height.

For unsupervised comparison of metabolomes t-distributed stochastic neighbor embedding (tSNE) was performed using the Rtsne package [49]. Perioperative changes in metabolites were identified by linear mixed effects modelling (LMM) with also included random effects (between patient variation), in addition to the fixed effects (different time points). The nlme packages was used for modelling within R [50].

Metabolites with FDR-corrected *p*-values < 0.05 were considered statistically significant for changes between T1–T8, unless specified otherwise. For subgroup analysis (e.g., T1–T3), *p*-values were not adjusted for multiplicity because of the small sample sizes.

All *p*-values were corrected for multiple testing and are given as FDR-corrected values, unless specified otherwise.

Since period T1–T3 represents the anesthetic period without ongoing surgical preparations and T3–T7 represents the joint period with anesthesia and ongoing surgery, a period with early onset metabolomic changes and one period with late onset metabolomic changes was defined. Metabolites with early onset concentration changes were defined as having a higher concentration change between T1 and T3 than within the whole observation period T1 to T8. Statistically this is represented by an absolute value of the coefficient of the regression analysis between T1–T3 being greater than between T1–T8 (|coefficient T1–T3| > |coefficient T1–T8|). Metabolites that did not increase strongest within the early period were defined as late onset changes. Multivariate analysis was performed to identify possible influences on plasma metabolites by the choice of anesthetic volatile (sevoflurane or desflurane) and administration of dexamethasone for PONV prophylaxis.

## SUPPLEMENTARY MATERIALS



## Abbreviations

3-IAA: 3-indoleaceitic acid
3-IPA: 3-indolpropionic acid
ASA: American Society of Anesthesiologists
CoA: Coenzyme A
COS: Centre for Organismal Studies (Heidelberg, Germany)
FA: fatty acid
FDR: false discovery rate
FIA-MS: flow injection analysis mass spectrometry
GFR: glomerular filtration rate
IDO: Indolamin-2,3-dioxygenase
IPMN: intraductal papillary mucinous neoplasm
LC-MS: Liquid Chromatography Mass Spectrometry
LOD: limit of detection
lysoPC: lysophosphatidylcholine
MRM: multiple reaction monitoring
MS: mass spectrometry
NYHA: New York Heart Association
PDAC: pancreatic ductal adenocarcinoma
PITC: phenyl isothiocyanate
PONV: postoperative nausea and vomiting
PPPD: pylorus preserving pancreaticoduodenectomy
PRBC: packed red blood cells
TDO: tryptophan-2,3-dioxygenase
TMAO: trimethylamine N-oxide
TRP: tryptophan
