# Supplementary material for: Perioperative changes in the plasma metabolome of patients receiving general anesthesia for pancreatic cancer surgery
Source: Oncotarget. 2021 May 11;12(10):996–1010. doi: 10.18632/oncotarget.27956 (PMC8121611; doi:10.18632/oncotarget.27956)
Supplement: Supplementary file 1 [file oncotarget-12-996-s001.pdf]

Perioperative changes in the plasma metabolome of patients receiving general anesthesia for pancreatic cancer surgery

SUPPLEMENTARY MATERIALS

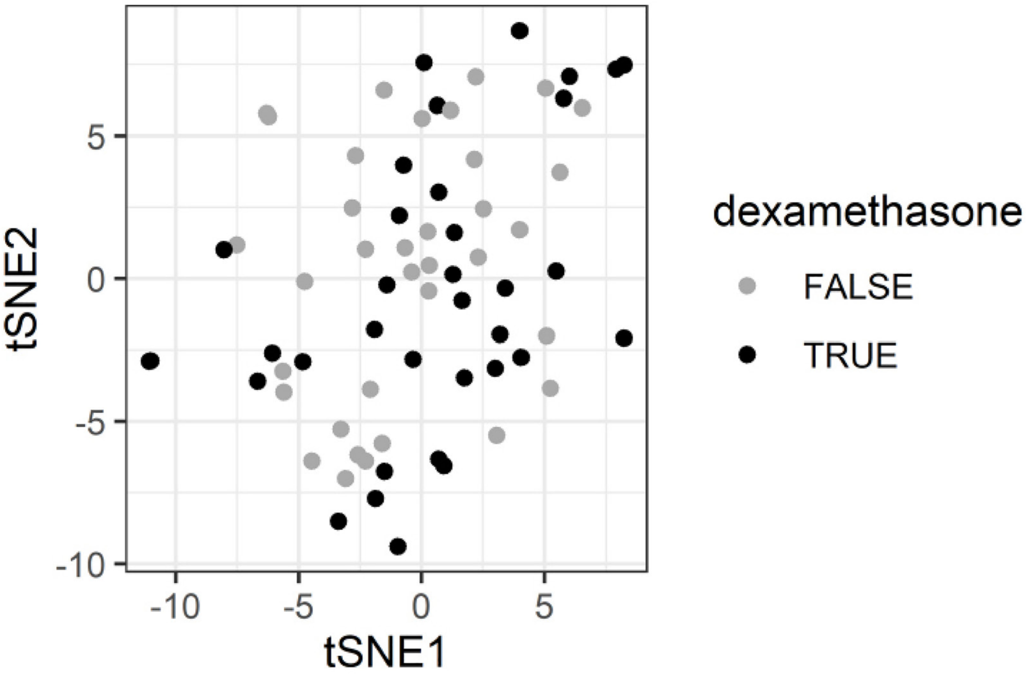

Supplementary Figure 1: Further tSNE plot for unsupervised comparison of the metabolome. Usage of dexamethasone had no systematic effect on the plasma metabolome. Metabolome data from all patients and all timepoints (80 samples) are depicted according to whether dexamethasone was used (black) or not (grey).

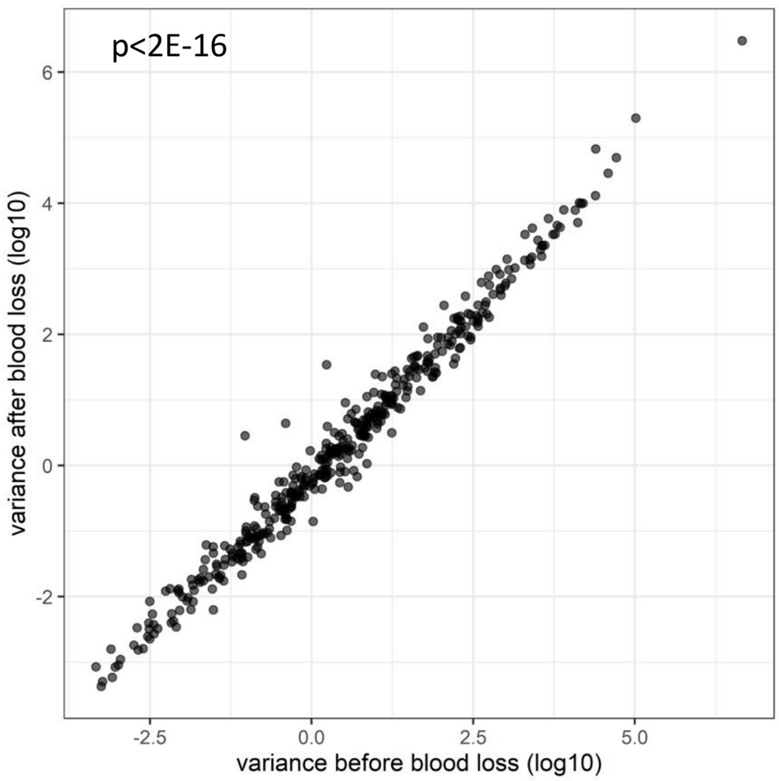

Supplementary Figure 2: Variance in plasma metabolite concentrations before and after major blood loss. Linear regression analysis revealed a high concordance between variances (p < 2E-16). Hence, the extent of plasma metabolite changes was not significantly different.

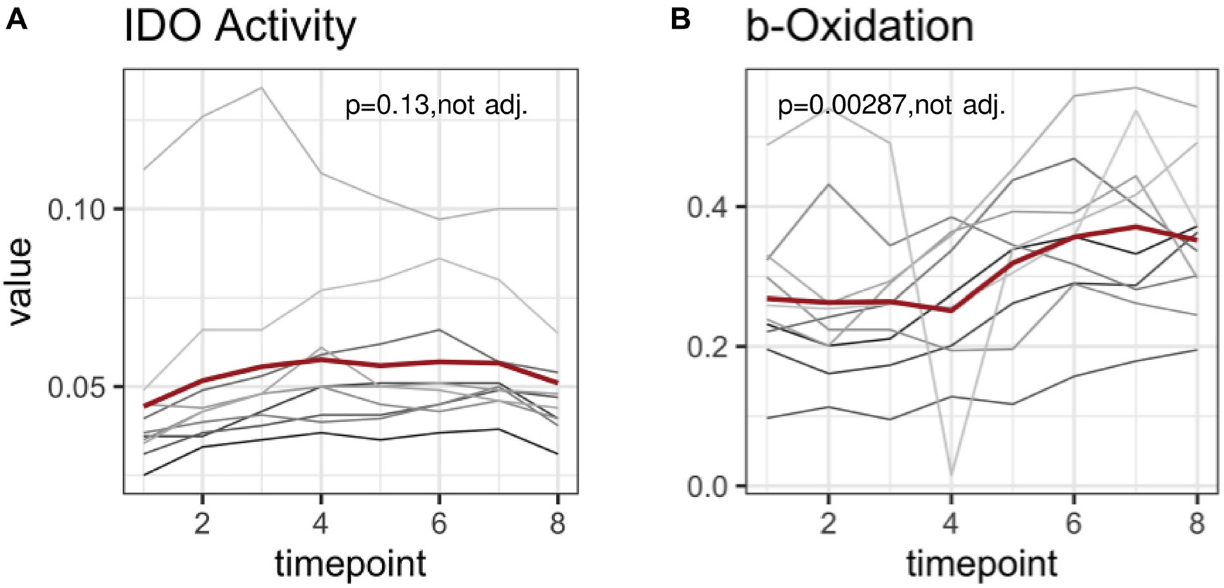

Supplementary Figure 3: Further metabolomic interpretation. Ratios of metabolites are depicted. Mean concentrations are depicted as a red line while individual courses of each patient are depicted in grey lines. *p*-values are not adjusted for multiple testing. (**A**) Ratio of [kynurenine]/[tryptophane] represents Indolamin-2,3-dioxygenase activity (IDO-activity). IDO-activity did not change significantly during observation period. (**B**) The ratio of propionyl carnitine plus acetyl carnitine to carnitine ([propionyl carnitine + acetyl carnitine]/[carnitine]), serves as an indicator for b-oxidation rates. B-oxidation increased during observation period but lost statistical significancy after adjusting for multiple testing.

Supplementary Table 1: List of significantly changing metabolites and MetaboINDICATORS™

Metabolites	class	*p*-val	*p*-adjust	coeff
**Carnitine**	Acylcarnitines	1,65E-06	9,30E-04	-1,60E+00
**Propionyl carnitine**	Acylcarnitines	2,88E-07	1,64E-04	-2,37E-02
**TMAO**	Amine Oxides	8,26E-05	4,40E-02	-5,81E-01
**Cysteine**	Amino acids	3,97E-05	2,15E-02	-4,30E+00
**Tryptophan**	Amino acids	1,08E-09	6,24E-07	-3,48E+00
**1-Methylhistidine**	Amino acids Related	8,85E-07	5,03E-04	-1,73E-01
**Kynurenine**	Amino acids Related	7,13E-10	4,14E-07	-1,18E-01
**t4-Hydroxyproline**	Amino acids Related	5,45E-06	3,05E-03	-4,27E-01
**Taurine**	Amino acids Related	9,07E-05	4,79E-02	2,98E+00
**Lactate**	Carboxylic Acids	4,73E-06	2,65E-03	1,27E+02
**FA(18:1)**	Fatty Acids	5,14E-05	2,77E-02	1,59E+01
**lysoPC a C16:0**	Glycerophospholipids	6,15E-05	3,29E-02	-3,59E+00
**lysoPC a C16:1**	Glycerophospholipids	1,55E-06	8,79E-04	-1,21E-01
**lysoPC a C18:0**	Glycerophospholipids	7,76E-05	4,14E-02	-8,59E-01
**lysoPC a C18:1**	Glycerophospholipids	1,18E-07	6,81E-05	-9,04E-01
**lysoPC a C18:2**	Glycerophospholipids	1,59E-05	8,78E-03	-9,06E-01
**lysoPC a C20:3**	Glycerophospholipids	3,27E-07	1,86E-04	-9,07E-02
**lysoPC a C20:4**	Glycerophospholipids	3,05E-06	1,71E-03	-2,99E-01
**3-Indoleaceitic acid**	Indoles Derivatives	2,21E-09	1,28E-06	-1,56E-01
**TG(16:0_38:7)**	Triacylglycerols	3,24E-05	1,77E-02	-4,43E-02
**TG(16:0_40:6)**	Triacylglycerols	2,28E-06	1,28E-03	-2,18E-01
**TG(16:0_40:7)**	Triacylglycerols	1,09E-06	6,16E-04	-1,98E-01
**TG(16:0_40:8)**	Triacylglycerols	3,81E-05	2,07E-02	-7,88E-02
**TG(17:1_34:3)**	Triacylglycerols	3,71E-05	2,02E-02	-3,70E-02
**TG(17:2_36:2)**	Triacylglycerols	2,63E-05	1,44E-02	-2,43E-02
**TG(18:1_34:1)**	Triacylglycerols	8,98E-05	4,76E-02	-2,27E+01
**TG(18:1_35:2)**	Triacylglycerols	9,27E-05	4,88E-02	-4,16E-01
**TG(18:1_38:6)**	Triacylglycerols	5,35E-05	2,87E-02	-1,68E-01
**TG(18:1_38:7)**	Triacylglycerols	7,89E-05	4,20E-02	-2,90E-02
**TG(18:3_34:0)**	Triacylglycerols	5,53E-06	3,08E-03	-1,04E-01
**TG(18:3_34:1)**	Triacylglycerols	9,18E-05	4,84E-02	-9,30E-01
**TG(20:1_32:1)**	Triacylglycerols	8,98E-05	4,76E-02	-6,92E-02
**TG(22:5_32:1)**	Triacylglycerols	3,64E-05	1,99E-02	-9,30E-02
**TG(22:5_34:1)**	Triacylglycerols	2,64E-06	1,49E-03	-3,94E-01
**TG(22:5_34:2)**	Triacylglycerols	2,10E-05	1,15E-02	-1,93E-01
**TG(22:6_32:0)**	Triacylglycerols	1,32E-05	7,29E-03	-1,76E-01
**TG(22:6_32:1)**	Triacylglycerols	5,76E-06	3,21E-03	-2,04E-01
**TG(22:6_34:1)**	Triacylglycerols	2,56E-07	1,46E-04	-7,32E-01
**TG(22:6_34:2)**	Triacylglycerols	1,10E-05	6,07E-03	-4,09E-01
**MC Deficiency (NBS)**	INDICATOR	3,93E-05	2,13E-02	1,87E-02
**DLD (NBS)**	INDICATOR	2,63E-07	1,51E-04	-1,17E-01

**Fischer Ratio**	INDICATOR	1,54E-10	8,98E-08	9,73E-02
**PKU (NBS)**	INDICATOR	4,14E-09	2,39E-06	-1,99E-02
**Ratio of Non-Essential to Essential AAs**	INDICATOR	5,99E-05	3,21E-02	-3,00E-02
**Sum of Sulfur-Containing AAs**	INDICATOR	8,46E-05	4,49E-02	-4,40E+00
**Cit Synthesis**	INDICATOR	1,70E-05	9,34E-03	-1,94E-02
**GABR**	INDICATOR	4,23E-08	2,44E-05	4,29E-02
**NO-Synthase Activity**	INDICATOR	2,79E-10	1,62E-07	-2,15E-02
**Pro Hydroxylation**	INDICATOR	2,71E-07	1,55E-04	-1,87E-03
**Taurine Synthesis**	INDICATOR	2,54E-07	1,46E-04	4,18E-02
**Sum of Carboxylic Acids**	INDICATOR	5,52E-06	3,08E-03	1,27E+02
**Ratio of Hex3Cer to Cer**	INDICATOR	4,69E-05	2,54E-02	-1,03E-02
**Ratio of DGs to FAs**	INDICATOR	7,84E-11	4,58E-08	-7,20E-03
**Sum of MUFAs**	INDICATOR	5,17E-05	2,78E-02	1,65E+01
**Ratio of TGs to FAs**	INDICATOR	1,40E-12	8,21E-10	-7,57E-01
**Sum of LCFA-LysoPCs**	INDICATOR	1,18E-05	6,55E-03	-6,82E+00
**Sum of LysoPCs**	INDICATOR	1,08E-05	5,98E-03	-6,86E+00
**Sum of MUFA-LysoPCs**	INDICATOR	5,55E-08	3,20E-05	-1,05E+00
**Sum of PUFA-LysoPCs**	INDICATOR	6,12E-06	3,40E-03	-1,30E+00
**Sum of SFA-LysoPCs**	INDICATOR	4,70E-05	2,54E-02	-4,52E+00

Abbreviations: TMAO: Trimethylamine N-oxide; FA: fatty acid; lysoPC: lysophosphatidylcholine; TG: triacylglyceride; MC: Multiple Carboxylase; DPD: Dihydrolipoamide Dehydrogenase Deficiency; PKU: Phenylketonuria; AA: Amino acid; Cit: Citrulline; GABR: Global Arginine Bioavailability Ratio; Pro: Proline; Hex3Cer: Trihexosylceramides; DG: Diglycerides; MUFA: Monounsaturated Fatty Acid; LCFA: Long-Chain Fatty Acid; PUFA: Polyunsaturated Fatty Acid; SFA: Saturated Fatty Acid.
